# Boron Partitioning Coefficient above Unity in Laser Crystallized Silicon

**DOI:** 10.3390/ma10020189

**Published:** 2017-02-16

**Authors:** Patrick C. Lill, Morris Dahlinger, Jürgen R. Köhler

**Affiliations:** Institute for Photovoltaics and Research Center SCoPE, University of Stuttgart, Pfaffenwaldring 47, 70569 Stuttgart, Germany; morris.dahlinger@ipv.uni-stuttgart.de (M.D.); juergen.koehler@ipv.uni-stuttgart.de (J.R.K.)

**Keywords:** solute trapping, rapid solidification, silicon, laser melting, boron doping

## Abstract

Boron pile-up at the maximum melt depth for laser melt annealing of implanted silicon has been reported in numerous papers. The present contribution examines the boron accumulation in a laser doping setting, without dopants initially incorporated in the silicon wafer. Our numerical simulation models laser-induced melting as well as dopant diffusion, and excellently reproduces the secondary ion mass spectroscopy-measured boron profiles. We determine a partitioning coefficient $k_{p}$ above unity with $k_{p}=1.25\pm 0.05$ and thermally-activated diffusivity $D_{B}$, with a value $D_{B}\left(1687\;K\right)\;=\;\left(3.53\;\pm \;0.44\right)\;\times \;10^{-4}$ cm${}^{2}$·s${}^{-1}$ of boron in liquid silicon. For similar laser parameters and process conditions, our model predicts the anticipated boron profile of a laser doping experiment.

## 1. Introduction

Pulsed laser melting of silicon (Si) has been a research topic since the 1960s [1]. Laser doping and annealing of implanted Si wafers are the two major applications, whose key features are the rearrangement and incorporation of dopants by diffusion in liquid Si. Due to the short time period of the energy input, the melting time of Si is in the range of several 100 ns for pulsed laser irradiation [2]. Considering the whole Si wafer, the thermal budget is minimal, and large temperature gradients between the liquid Si and the surrounding solid phase exist. Owing to the steep temperature gradients, re-solidification after termination of the laser pulse proceeds very quickly, with liquid/solid interface velocities $v_{i}$ of several m/s [3]. The rapid solidification leads to non-equilibrium crystallization at the advancing phase interface, causing dopant concentrations in the just-formed Si solid which do not match their equilibrium values.

The partitioning (or segregation) coefficient $k_{p}=C_{s}/C_{l}$ commonly denotes the ratio of dopant concentrations in the Si melt, $C_{l}$, and crystal, $C_{s}$, directly at the liquid/solid interface. For sufficiently small solidification (interface) velocities $v_{i}$, $k_{p}$ approaches its equilibrium value $k_{eq}$, which is $k_{eq}<<1$ for most impurities in Si, but $k_{eq}=0.8$ for boron (B) [4,5], causing segregation of B into the melt. Due to the localized heat input during laser melting of a Si wafer, large temperature gradients between the melt and the surrounding crystal cause high interface velocities $v_{i}$, which can completely suppress segregation of B atoms into the melt. This process is commonly termed “solute trapping” [6] ($k_{p}≃1$ for complete solute trapping), and has been reported for various impurities in Si [4,7,8,9,10,11,12].

Several approaches to modeling the physical mechanisms leading to solute trapping at the advancing solidification exist in literature. An extensive listing can be found in the review of Sobolev [13] and the references therein. For the purpose of the discussion in this paper, these models can be divided into two classes, depending on whether they assume a sharp [10,14,15,16] or a diffusive (continuous) [17,18] interface between the growing solid and the liquid phase during solidification.

However, the solute trapping mechanism alone cannot explain the distinct B accumulation (pile-up) at the maximum melt depth, which was observed in several investigations on laser melt annealing of Si wafers with implanted B profiles [19,20,21,22,23]. Additionally, experiments [24,25] with B sources deposited on top of a Si wafer and repeated laser melting cycles (e.g., in a laser doping setting) also yielded dopant profiles exhibiting B accumulation effects.

The physical mechanisms causing the measured B accumulation are up to now a subject of discussion in literature. Monakhov et al. assumed that excess vacancies formed during laser treatment inside the solid part of the Si wafer adjacent to the maximum melt depth caused the boron pile-up [20]. For corroboration of their excess vacancy theory, they referred to an investigation which reported a vacancy accumulation at the maximum melt depth for repeated laser melting [26]. In contrast, Ong et al. attributed the redistribution of B to the recrystallization process. They associated the boron pile-up formation with different recrystallization transients and respective varying segregation (partitioning) coefficients $k_{i}$ (e.g., $k_{p}$) at the moving liquid/solid interface [19], thus employing different values for $k_{i}$ depending on the interface position. Furthermore, the most recent model applied two-state diffusion in liquid Si to establish an adsorptive interface region between the liquid and solid phase which induces the B accumulation. Local bonding fluctuation in liquid Si was identified as the underlying physical mechanism [21,22,27]. However, while the models in these works indeed reproduce the reported respective experimental results, they cannot give a particular value of the partitioning coefficient for a specific solidification velocity, as is possible with the solute trapping models [10,14,15,16,17,18].

In principle, either within the framework of a Monte Carlo (MC) [28] or a phase-field (PF) [17] model, a partitioning coefficient above unity can be obtained when an adsorptive interface is assumed. The assumption of a continuous interface during rapid solidification of Si—in contrast to the sharp interface models—is further supported by studies [29,30], indicating that excess vacancies generated during the rapid re-solidification are connected with the formation of a distinct interface region between the liquid and solid phase.

In the present investigation, we observe a distinct B pile-up in a laser doping setting, and determine a partitioning coefficient $k_{p}$ by means of a numerical model which reproduces the experimental boron profiles remarkably well with a value of $k_{p}=1.25$, which is constant with melt depth, pulse energy, and number of laser scans. Our approach offers a simple and straightforward procedure to determining the partitioning coefficient from experimental results.

## 2. Experimental Section

We used (100)-oriented *n*-type float zone (FZ) silicon wafers with thickness d=290μm and resistivity ρ=0.5Ω·cm. Prior to sputter deposition of a thin (<1 nm) pure boron layer, a short dip in diluted hydrofluoric acid removes the native oxide of the wafer. We estimated the boron layer thickness from the sputtering parameters, because we have no means for a direct determination. A frequency doubled Nd:YAG laser emitting at wavelength λ=532 nm with a pulse repetition rate f=10kHz and a full pulse duration at half maximum (FDHM) $t_{p}=42\;$ns scanned the surface of the Si wafer.

**Figure 1:**
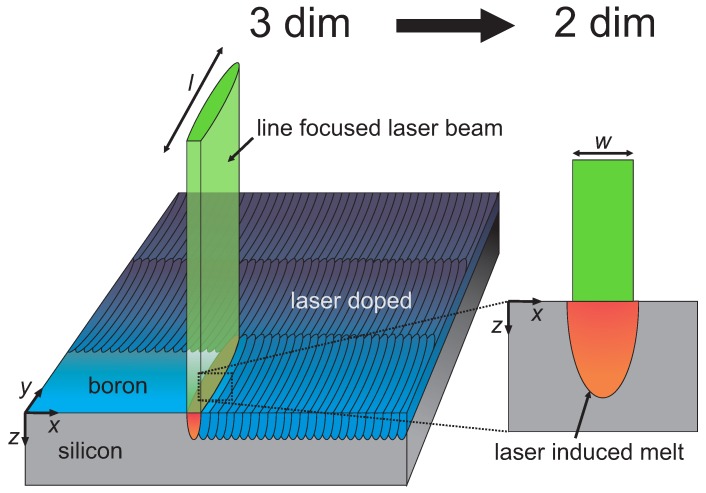
Sketch of the laser doping process. A line-shaped pulsed laser emitting at wavelength λ=532nm scans the wafer surface with a Gaussian intensity profile with full width at half maximum w=7μm along the short axis (*x*-direction) and a l=800μm top-hat profile in the long axis (*y*-direction). Due to the line-shaped laser beam with l>>w, the gradient of the laser intensity in the *y*-direction is much smaller than in the *x*-direction. Hence, this justifies the reduction into two dimensions to numerically solve the heat transport and diffusion equation.

Figure 1 presents the laser doping process. An optical setup generates a line-shaped laser beam featuring a Gaussian intensity profile with full width at half maximum (FWHM) w=7μm in *x*-direction and top-hat profile with length l=800μm in *y*-direction. We determined the pulse duration and beam diameter as described in Reference [31].

To adjust the laser pulse energy $E_{p}$ while ensuring constant temporal pulse shape and pulse duration as well as minimal pulse-to-pulse energy variation, we used a combination of a λ/2 wave plate and a polarizing beam splitter cube, with the laser operating at constant power. Prior to secondary ion mass spectrometry (SIMS) measurements, a thin layer of amorphous silicon (20nm) was sputter-deposited on top of the samples to ensure that equilibrium conditions during SIMS measurement were reached before the actual B profile was recorded. The onset of the wafer was determined by the carbon surface peak and change in Si signal (not shown here).

In order to fabricate doped areas for sheet resistance and SIMS measurements, the laser system raster scanned the wafer surface (also schematically depicted in Figure 1) with a scanning speed $v_{l}\;=\;32\;$mm/s along the *x*-direction (corresponding to a pulse overlap of ≈50%), while the 800μm-wide laser traces overlap 50μm in the *y*-direction. To investigate the diffusion process of B in liquid Si, we varied the number of subsequent laser scans $N_{s}=1$, 2, 5, 10, 20, 30, and 40 at constant pulse energy $E_{p}$ for each individual laser processed area. We fabricated laser processed areas using three different pulse energies $E_{p1}=192\;\mu$J, $E_{p2}=237\;\mu$J, and $E_{p3}=264\;\mu$J, corresponding to laser fluences $H_{p1}=2.00\;$J·cm${}^{-2}$, $H_{p2}=2.47\;$J·cm${}^{-2}$, and $H_{p3}=2.75\;$J·cm${}^{-2}$, with the beam area defined for $1/e^{2}$ of the peak intensity.

## 3. Numerical Simulation

Our simulation is based on the finite-volume numerical model presented in Reference [32], transferred to a two-dimensional Cartesian coordinate system. The use of a line-focused laser beam (l>>w) resulted in much smaller gradients of the laser intensity, and thus temperature in the *y*-direction compared to in the *x*-direction. This feature justifies modeling the experiment as a two-dimensional (x,z) system, with the *x*-direction parallel to the short laser axis and the *z*-direction representing the depth inside the wafer. Diffusion of B in Si is included into the finite-volume method to solve the heat transport and diffusion equation as described in Reference [31]. Compared with the model of Köhler et al. [31], one noteworthy modification is a narrower temperature interval ΔT=10K to account for over-heating during melting and under-cooling while the Si re-crystallizes [3], because of lower liquid/solid interface velocities $v_{i}$ due to longer laser pulse durations $t_{p}$ in this work.

In contrast to numerous experiments exploring the effects of multiple laser pulses on implanted B in crystalline Si [19,20,21,22,23,33], there is no initial doping profile present in this investigation, because we used a thin sputter-deposited B layer on top of the wafer as dopant source. To model this source, a finite-volume grid element outside the Si surface acts as a finite dopant source [34], from which B atoms diffuse into the first Si element during melting. As long as the Si remains liquid, in-diffusion of B occurs according to the concentration gradient between the B dopant source and the first Si element. For subsequent laser pulses, the B concentration changes only through in-diffusion into Si, because material conservation is implied.

We assumed no alteration of the dopant source during the laser irradiation, especially no ablation and re-condensation of dopant atoms, because of the very thin B precursor layer (below 1nm) and the high melting temperature $T_{m}^{B}=2348$ K >> $T_{m}^{Si}=1687$ K of B compared with Si [35]. Possibly, all B atoms available for doping enter the liquid Si during the first melting cycle and an ultra-thin B/Si compound acts as the dopant source for the subsequent melting events. Nevertheless, we have no means of investigating the properties of this dopant source in full detail, and put the focus of this paper on the evolution of the dopant profiles inside the sample for repeated laser melting and recrystallization cycles. Although the exact physical nature of the doping mechanism from this ultra-thin source layer is not completely understood, the simulations match the experimental results very well. The comparison (see next section) of measured sheet resistances and calculated dopant doses supports the assumption of a finite dopant source without dopant loss for increasing number of laser scans.

For the comparison of our model with the experiments, we simulated a single laser scan line with six pulses separated by a distance Δx=3.2μm. To account for the positioning error of our experimental setup between two subsequent (repeated) scans, the starting point of each individual laser scan varied randomly around the set point, with a maximum deviation of ±Δx/2. The model also includes the pulse energy $E_{p}$ (laser power) fluctuations of the laser by varying $E_{p}$ for each laser pulse randomly around the set value (energy deviation within 3σ is ±3%). In a manner similar to Köhler et al. [31], we tuned the pulse energy in the numerical calculations in such a way that the simulated concentration depths fit the SIMS measured profiles for $N_{s}=30$ and 40, because small uncertainties in the pulse energy can lead to significant uncertainty in surface temperature, melt duration, and especially melt depth.

The concentrations $C_{l}$ and $C_{s}$ at the liquid/solid-interface are corrected for the effect of solute trapping at the moving interface in a mass-conserving manner at every time step Δt of the simulation,

(1) $$\begin{array}{cc} C_{l}^{cor.} & =C_{l}+\frac{D_{B}\Delta t}{\Delta z^{2}}\left[\frac{\left(1-k_{p}\right)C_{l}}{k_{p}}-\frac{\left(1+k_{p}\right)C_{s}}{k_{p}}\right] \end{array}$$

(2) $$\begin{array}{cc} C_{s}^{cor.} & =C_{s}-\frac{D_{B}\Delta t}{\Delta z^{2}}\left[\frac{\left(1-k_{p}\right)C_{l}}{k_{p}}-\frac{\left(1+k_{p}\right)C_{s}}{k_{p}}\right] \end{array}$$

where the partitioning coefficient $k_{p}$ together with the diffusivity $D_{B}$ and grid spacing Δz yield the “partitioning coefficient corrected” concentrations $C_{l}^{cor.}$ and $C_{s}^{cor.}$, respectively.

## 4. Results

Four-point probe and SIMS measurements determined the sheet resistance $R_{sh}$ and B profile of the laser irradiated areas. We determined a scaling factor by comparing $R_{sh}^{calc}$ computed by the PV Lighthouse [36] sheet resistance calculator from the SIMS boron profiles with the measured $R_{sh}$ values for each laser area. Assuming the whole B profile to be electrically active, as a high activation ratio for laser annealed B is known from literature [33], we linearly scaled the SIMS profiles with this scaling factor in a way that the calculated $R_{sh}^{calc}$ matched the measured values $R_{sh}$.

Table 1 presents the sheet resistances $R_{sh}$ for all laser processing parameters. At each of the three different pulse energies $E_{p1}=192\;\mu$J, $E_{p2}=237\;\mu$J, and $E_{p3}=264\;\mu$J, no trend of increasing $R_{sh}$ values with increasing number of subsequent laser scans $N_{s}$ exists.

Table 2 shows the calculated (from the unscaled SIMS profiles) dopant doses $B_{dop}^{calc}$ for all laser processing parameters. The fundamental assumption that no dopant loss occurs in our experiments is further supported by the fairly constant dose for all laser parameters; note that the imposed dose for the simulation was $B_{dop}^{simul}=1.3\times 10^{15}\;$cm${}^{-2}$.

This shows that—unlike in the case of phosphorous [31]—no significant out-diffusion occurred at the surface of the Si wafer for repeated laser scanning. Thus, it is justified to refrain from including out-diffusion into our model.

For the simulations, we used two fitting parameters—the partitioning coefficient $k_{p}$ and the “pre-factor” $D_{0}$ of the thermally activated boron diffusivity $D_{B}=D_{0}\times \exp\;\left[-E_{a}/\left(k_{B}T\right)\right]$ in liquid silicon [37]. Whereby *T* denotes the temperature, $E_{a}=119$ meV the activation energy (taken from Reference [37]) and $k_{B}$ is the Boltzmann constant. Due to the fact that the values of $D_{B}$ in solid and liquid state differ by seven orders of magnitude [37], the diffusivity of B in solid Si is set to zero in the simulation.

**Figure 2:**
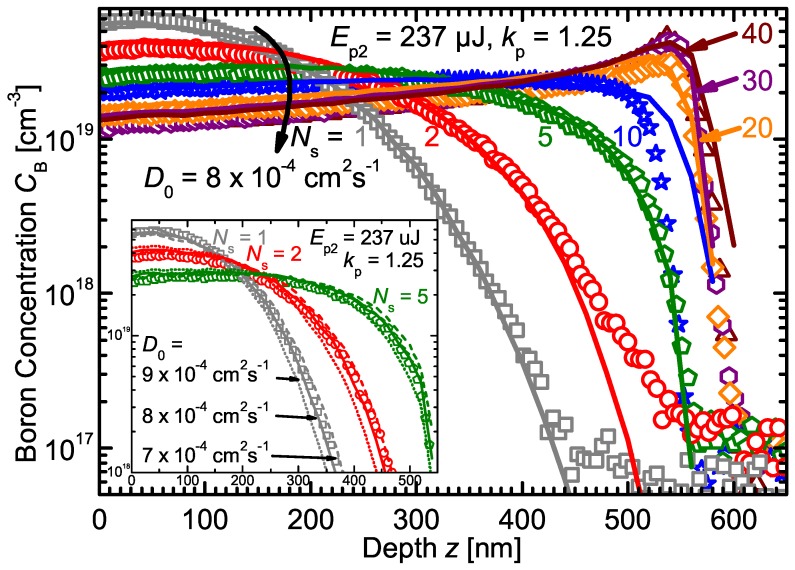
SIMS boron profiles (data points) of silicon wafers irradiated with varied number of laser scans $N_{s}$ for constant laser pulse energy $E_{p2}\;=\;237\;\mu$J. Numerical simulations (lines) with a diffusivity $D_{B}=\;8\;\times \;10^{-4}\;{\mathrm{cm}}^{2}$·$s^{-1}\times \exp\;\left[-E_{a}/\left(k_{B}T\right)\right]$ and partitioning coefficient $k_{p}=1.25$ yield the best fits to the SIMS data. The inset shows the influence of varied diffusivity on the shape of the simulated profiles for small number of laser scan repetitions.

Figure 2 shows the experimental B profiles resulting from a varied number of laser scans $N_{s}=1$, 2, 5, 10, 20, 30, and 40 with constant pulse energy $E_{p2}\;=237\;\mu$J. It is apparent from the B profiles that a boron pile-up at the maximum melt depth $m_{d2}=550$ nm develops with increasing number of laser scans, which was also previously reported for implanted B [19,20,21,22]. Simulations with $k_{p}=1.25$ and $D_{0}=8\times 10^{-4}$ cm${}^{2}$·s${}^{-1}$ reproduce the experimental B profiles well for all number of scans $N_{s}$.

The inset of Figure 2 illustrates how the B diffusivity $D_{B}$ influences the simulated profiles. The overall B concentration gradient throughout the recrystallized Si (until just ahead of the maximum melt depth) decreased with increasing number of laser scans until $N_{s}=10$. At higher $N_{s}$, a gradient sign conversion accompanied by the B pile-up formation resulted in increasing gradients with increasing laser scans for $N_{s}>10$. Adjacent to the surface dopant source, $D_{B}$ in conjunction with the concentration gradient determines the rapidity of the B incorporation. The concentration profiles became relatively flat in the near-surface region with higher melting cycles, and thereby the diffusivity’s influence on the final B profile subsides. To work out the influence of the diffusivity alone, the pulse energy was kept constant for all simulations depicted in the inset (no pulse energy randomization and thus no melt depth variation). Comparison of the measured and simulated B profiles shows for the three scans that simulations with $D_{0}=\left(8\pm 1\right)\times 10^{-4}$ cm${}^{2}$·s${}^{-1}$ reproduced the experimental data most accurately. Therefore, we determined the diffusivity of B in liquid Si as $D_{B}\left(1687\;K\right)=\left(3.53\pm 0.44\right)\times 10^{-4}$ cm${}^{2}$·s${}^{-1}$. Although this value is of the same order of magnitude as the boron diffusivity in liquid silicon reported in literature [5,37,38], it is higher by a factor of up to three. A possible reason for this discrepancy could be the large temperature gradient present in the laser molten Si. The temperature close to the sample surface can be significantly larger than the Si melting temperature; thus, the determined diffusivity is technically averaged over this temperature interval ($T_{max}\;-\;T_{m}^{Si}$).

**Figure 3:**
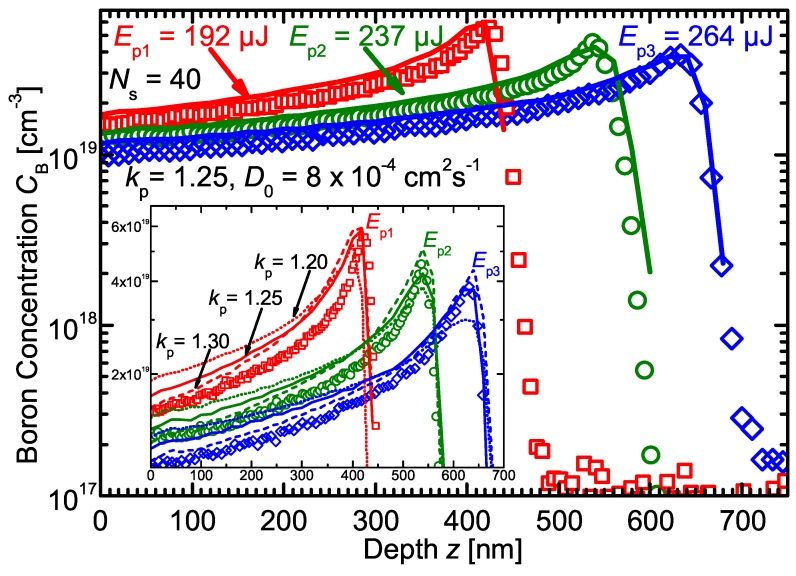
SIMS boron profiles (data points) of silicon wafers irradiated with varied laser pulse energies $E_{p1}=192\;\mu$J, $E_{p2}=237\;\mu$J, and $E_{p3}=264\;\mu$J for constant number of laser scans $N_{s}=40$. Numerical simulations (lines) with a diffusivity $D_{B}=8\;\times \;10^{-4}\;{\mathrm{cm}}^{2}$·$s^{-1}\times \exp\;\left[-E_{a}/\left(k_{B}T\right)\right]$ and partitioning coefficient $k_{p}=1.25$ yield the best fits to the SIMS data. The inset shows the influence of varied partitioning coefficients on the shape of the simulated profiles.

Figure 3 presents the B profiles laser irradiated with pulse energies $E_{p1}=192\;\mu$J, $E_{p2}=237\;\mu$J, and $E_{p3}=264\;\mu$J for a constant number of scans $N_{s}=40$. The pulse energy sets the maximum melting depth, and the boron pile-up occurred for all three pulse energies at the respective melting depths $m_{d1}=430$ nm, $m_{d2}=550$ nm, and $m_{d3}=640$ nm. Increased melt depth $m_{d}$—due to higher pulse energy $E_{p}$—leads to a less pronounced boron pile-up at the maximum melt depth.

Although impurity diffusion is much faster in liquid Si compared to the solid phase, the B atoms need some time to reach a given depth, which is also clearly observable in Figure 2 where the concentration profile depth does not reach the maximum melt depth until the tenth laser scan. Whereas the pulse duration is constant for the different pulse energies, the melt duration increases with increasing $E_{p}$ due to the higher heat input. The sheet resistance results together with the calculated doses prove that the same amount of B atoms is incorporated into the Si upon recrystallization for all pulse energies, whereby longer melt durations (and larger melt depths) for increasing pulse energy shift the B profile to lower concentrations. Again, the simulated and experimental B profiles agree well for all of the investigated pulse energies $E_{p1}=192\;\mu$J, $E_{p2}=237\;\mu$J, and $E_{p3}=264\;\mu$J at a constant number of scans $N_{s}=40$, which supports the selection of $k_{p}=1.25$ and $D_{0}=8\times 10^{-4}$ cm${}^{2}$·s${}^{-1}$.

The inset of Figure 3 presents a zoom of the boron pile-up region, which demonstrates how an increasing partitioning coefficient $k_{p}$ results in a steeper profile slope and a more pronounced B accumulation. For all three pulse energies $E_{p}$, a partitioning coefficient of $k_{p}=1.20$ results in a too-marginal pile-up and shallow slope, whereas for $k_{p}=1.30$, the pile-up is exaggerated and the slope is too steep. A partitioning coefficient $k_{p}=1.25$ reproduces the slope and extent of the pile-up well. It is quite clear that the correct value for the partitioning coefficient lies close to $k_{p}=1.25$. Thus, within the framework of our experiment, the non-equilibrium partitioning coefficient of B in liquid Si is $k_{p}=1.25\pm 0.05$.

## 5. Discussion

In the framework of the sharp interface models, the maximum possible partitioning coefficient is unity, although they deviate on the issue if complete solute trapping ($k_{p}=1$) is reached at a finite interface speed $v_{i}$ or only approximated with increasing speed of solidification ($v_{i}\;\to \;\infty \Rightarrow k_{p}\;\to \;1$). Therefore, apparently only the diffusive (continuous) interface approaches could serve as the foundation for an extended solute trapping model accounting for $k_{p}$ values above unity which are attributed to the B pile-up in laser-crystallized Si.

Consequently, the adsorptive interface hypothesis proposed by a various authors [21,22,27] is most likely the approach which can explain our experimental results with the ascertained partitioning coefficient above unity $k_{p}=1.25$. Although Ahmad et al. [18] discuss the fact that their PF model requires an advancement to cover situations with significant interface adsorption, the $k_{p}>1$ case has been reported within the PF model framework [17]. Thus, the PF model appears to be the most promising approach to incorporate a diffusive/absorptive interface and model the B accumulation during rapid solidification. Nevertheless, a predictive model for rapid solidification that includes an adsorptive interface and correctly reproduces the B pile-up with an overall constant partitioning coefficient (for a distinct solidification speed) is yet to be developed.

Such an extended model could be tested against our experimental data, and additional investigations with different laser pulse duration and/or sample background temperature can complement the data for different solidification speeds. Our method demonstrates that the familiar B pile-up in laser crystallized silicon is excellently reproduced with a partitioning coefficient above unity $k_{p}=1.25$. In comparison with the previous studies on the the matter, our approach requires less assumptions (no interface with appropriate properties, no varying values of the partitioning coefficient or vacancy distributions) to determine the overall $k_{p}$ of B in laser crystallized Si. Moreover, as our procedure utilizes a laser doping setting, no sophisticated ion implantation prior to the first laser pulse is necessary. Thus, it offers a simple, straightforward procedure to obtain the $k_{p}$ value of a given experimental situation. Therefore, supplementing data (different $k_{p}$ for varied $v_{i}$) could be obtained readily once an extended solute trapping model with adsorptive interface is to be tested against B profiles in laser-crystallized Si.

Although our numerical model is not predictive insofar as it does not yield a partitioning coefficient for any solidification speed and given material parameters (dopant species, substrate material, laser wavelength, and corresponding optical absorption coefficient, etc.), it nonetheless yields the anticipated boron profile for comparable laser parameters and process conditions. This feature can help to minimize the number of required diffusion experiments during a dopant profile optimization study. Our measurements together with a boron partitioning coefficient above unity indicate the requirement for an extended model to describe the boron pile-up within the solute trapping framework.

## 6. Conclusions

In conclusion, we have determined a diffusivity $D_{B}\left(1687\;K\right)=\left(3.53\pm 0.44\right)\times 10^{-4}$ cm${}^{2}$·s${}^{-1}$ and a partitioning coefficient $k_{p}=1.25\pm 0.05$ of boron in liquid silicon for pulsed laser melting. A thin sputter-deposited layer of pure B at the surface of FZ Si wafers serves as finite dopant source for laser doping, thus establishing the starting profile for the investigation of B redistribution during repeated laser melting. Our numerical model correctly reproduces the extent of the boron built-up at the maximum melt depth and also describes its dependence on number of laser scans and pulse energy. The diffusivity and partitioning coefficient are the only fitting parameters needed to excellently reproduce the experimentally-measured SIMS profiles for varied number of laser scans $N_{s}=1$, 2, 5, 10, 20, 30, and 40 at pulse energies $E_{p1}=192\;\mu$J, $E_{p2}=237\;\mu$J, and $E_{p3}=264\;\mu$J. Our simulation implements a straightforward approach to model the boron diffusion and accumulation during laser melting of Si. In the context of dopant profile optimization studies, our numerical model can help to minimize the number of required laser doping runs, because it predicts the anticipated boron profile of a laser doping experiment if similar laser parameters and process conditions are applied.

## Figures and Tables

**Table 1:** Four-point probe measured sheet resistance $R_{sh}$ of areas treated by repeated laser scans for three laser pulse energies $E_{p}$. No dopant loss (e.g., out-diffusion) occurs, because no significant change of $R_{sh}$ is observable for increasing $N_{s}$.

Laser Scans	Sheet Resistance $R_{\mathrm{sh}}$ (Ω/sq)		
$N_{s}$	$E_{p1}\;=\;192\;\mu$J	$E_{p2}\;=\;237\;\mu$J	$E_{p3}\;=\;264\;\mu$J
1	90 ± 1.4	95 ± 1.5	85 ± 1.3
2	89 ± 3.6	97 ± 1.5	83 ± 1.5
5	84 ± 1.4	92 ± 1.6	85 ± 2.4
10	86 ± 0.9	88 ± 1.3	83 ± 2.0
20	90 ± 1.2	93 ± 1.2	89 ± 0.9
30	91 ± 1.5	91 ± 1.2	90 ± 1.3
40	96 ± 1.8	93 ± 0.8	88 ± 1.4

**Table 2:** Calculated dose $B_{dop}^{calc}$, obtained through integration of the (unscaled) secondary ion mass spectrometry (SIMS)-measured boron concentration profiles, of areas treated by repeated laser scans for three laser pulse energies $E_{p}$. No dopant loss (e.g., out-diffusion) occurs, because $B_{dop}^{calc}$ is roughly constant for all combinations of $E_{p}$ and $N_{s}$.

Laser Scans	Calculated Dose $B_{\mathrm{dop}}^{\mathrm{calc}}$ (cm${}^{-2}$)		
$N_{s}$	$E_{p1}\;=\;192\;\mu$J	$E_{p2}\;=\;237\;\mu$J	$E_{p3}\;=\;264\;\mu$J
1	1.02 $\times \;10^{15}$	1.38 $\times \;10^{15}$	1.08 $\times \;10^{15}$
2	1.29 $\times \;10^{15}$	1.18 $\times \;10^{15}$	1.36 $\times \;10^{15}$
5	1.28 $\times \;10^{15}$	1.20 $\times \;10^{15}$	1.38 $\times \;10^{15}$
10	1.56 $\times \;10^{15}$	1.30 $\times \;10^{15}$	1.63 $\times \;10^{15}$
20	1.30 $\times \;10^{15}$	1.54 $\times \;10^{15}$	1.32 $\times \;10^{15}$
30	1.28 $\times \;10^{15}$	1.65 $\times \;10^{15}$	1.35 $\times \;10^{15}$
40	1.11 $\times \;10^{15}$	1.23 $\times \;10^{15}$	1.10 $\times \;10^{15}$
